# Cloning and Functional Verification of Genes Related to 2-Phenylethanol Biosynthesis in *Rosa rugosa*

**DOI:** 10.3390/genes9120576

**Published:** 2018-11-26

**Authors:** Lixia Sheng, Yuqian Zeng, Tiantian Wei, Min Zhu, Xuemin Fang, Xiaoyu Yuan, Yunjian Luo, Liguo Feng

**Affiliations:** College of Horticulture and Plant Protection, Yangzhou University, Yangzhou 225009, China; lxsheng@yzu.edu.cn (L.S.); zengyq@cloudhealth99.com (Y.Z.); tiantianwei821@163.com (T.W.); 18752784267@163.com (M.Z.); FANGXUEMIN48@163.com (X.F.); yuanxiaoyu199405@163.com (X.Y.); yjluo@yzu.edu.cn (Y.L.)

**Keywords:** *Rosa rugosa*, floral scent, essential oil, 2-phenylethanol biosynthesis, gene functional verification

## Abstract

In China, *Rosa rugosa* is cultivated as a source of natural perfumes. Rose essential oil is known as “liquid gold”, given its high economic and health value. 2-phenylethanol accounts for more than 10% of the total mass fraction of the essential oil derived from *R. rugosa*. The regulatory mechanisms underlying 2-phenylethanol metabolism in *R. rugosa*, however, remain unclear. In this study, *RrAAAT* and *RrPPDC1*, two genes related to 2-phenylethanol synthesis, were cloned from *R. rugosa.* Expression analysis revealed that *RrAAAT* and *RrPPDC1* were highly expressed in rose flowers in the full opening and withering stages, and in calyxes. The overexpression vectors of *RrAADC*, *RrAAAT*, and *RrPPDC1* were established and transformed into *Petunia hybrida* via *Agrobacterium*-mediated genetic transformation. Results demonstrated that the overexpression of *RrAADC* and *RrAAAT* increased the 2-phenylethanol content of transgenic petunia flowers. The results of this study provide a basis for the introduction of genes related to 2-phenylethanol synthesis into roses to increase the 2-phenylethanol content of rose essential oil.

## 1. Introduction

*Rosa rugosa* is an economically useful ornamental plant that belongs to the Rosaceae family. It has been cultivated for more than 1300 years in China. Rose essential oil is extracted from rose flowers, and is known as “liquid gold”. It is widely used in high-end cosmetics and aromatherapy. In Bulgaria, France, Turkey, Morocco, and other European countries, rose essential oil is extracted mainly from the flowers of *Rosa damascena* or *Rosa centifolia*, which have been recently introduced to China. The morphological characteristics and floral headspace compounds of these flowers, however, differ from those of the traditional Chinese rose [1].

Rose essential oil contains numerous important aromatic components, including 2-phenylethanol, which is an important reference standard that is used for evaluating the quality of rose essential oil. 2-Phenylethanol accounts for more than 10% of the total mass fraction of traditional Chinese rose essential oil derived from *R. rugosa* [2,3]. The demand for 2-phenylethanol has increased, given the extensive use of this compound in food, cosmetics, tobacco, toiletries, and antimicrobial and medical supplies. Pure natural 2-phenylethanol extracted from roses is popular among consumers because of its nontoxicity and sensory qualities. Therefore, elucidating the mechanism of 2-phenylethanol biosynthesis in traditional Chinese roses at the molecular level will provide a basis for increasing the 2-phenylethanol content of *R. rugosa* and improving the yield and quality of rose essential oil through molecular breeding and genetic engineering.

**Figure 1:**
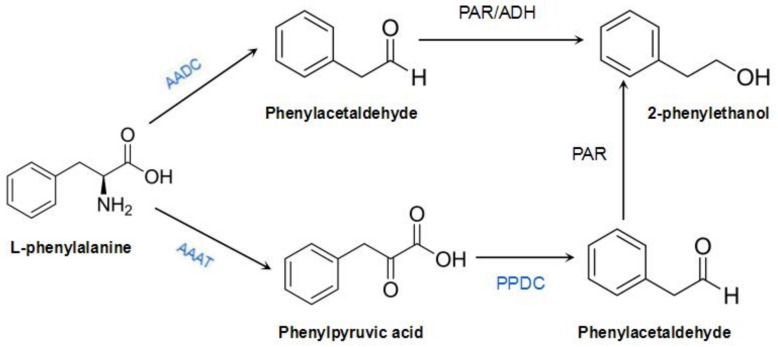
Biosynthesis pathway of 2-phenylethanol in *Rosa* species. PAR: phenylacetaldehyde reductase; ADH: alcohol dehydrogenase.

In roses, phenylpyruvic acid or phenylacetic acid is an intermediate in the 2-phenylacohol synthesis pathway, which has yet to be fully elucidated [4]. Isotope-labeling studies on the anabolic pathways of 2-phenylacohol in *R. damascena* and *Rosa* × *hybrida* “Hoh-Jun” showed that l-phenylalanine is first converted into phenylacetaldehyde, and then reduced to 2-phenylacohol [5,6]. Sakai et al. further speculated that the key enzyme that mediates the direct conversion of l-phenylalanine into phenylacetaldehyde in roses is l-aromatic amino acid decarboxylase (AADC) and that the key enzymes involved in the conversion of phenylacetaldehyde into 2-phenylacohol are phenylacetaldehyde reductase (PAR) and alcohol dehydrogenase (ADH) [7]. In addition, they speculated that several pathways are involved in 2-phenylacohol synthesis, in which phenylpyruvate is an intermediate, and the gene encoding aromatic amino acid transaminase (AAAT) is the key gene [8]. Another 2-phenylethanol synthetic pathway in rose was discovered through the cloning of *RhAAAT* and the gene encoding phenylpyruvate decarboxylase (*RhPPDC*), from the perpetually blooming rose cultivar *R. hybrida* “Yves Piaget”. In this pathway, l-phenylalanine generates phenylpyruvic acid in the presence of AAAT, but is not directly synthesized into phenylacetaldehyde. Phenylacetaldehyde production is then catalyzed by PPDC. PAR reduces phenylacetaldehyde to 2-phenylethanol. This pathway is influenced by seasonality, and it is activated only during summer [9,10]. However, so far, the synthetic and regulatory mechanisms of 2-phenylethanol metabolism in R. rugosa remain unclear. *R. rugosa, R. hybrida*, and *R. damascena* belong to the genus *Rosa*, and they likely share similar 2-phenylethanol biosynthetic pathways [9] (Figure 1). We had previously cloned *RrAADC* from *R. rugosa* and characterized its expression patterns [11]; in the present study, we continue to clone genes related to 2-phenylethanol synthesis in the traditional Chinese rose variety *R. rugosa* ‘Tanghong’, investigate their spatiotemporal expression characteristics, and verify the functions of the three genes (marked in blue in Figure 1). Our findings will provide a foundation for further research on the molecular mechanism underlying the regulation of 2-phenylethanol synthesis in the traditional Chinese rose.

## 2. Materials and Methods 

### 2.1. Cloning of the RrAAAT and RrPPDC1 Genes 

#### 2.1.1. RNA Extraction and Purification

The traditional Chinese rose cultivar, *R. rugosa* ‘Tanghong’ was used as experimental material. Total RNA was isolated from the petals of *R. rugosa* flowers in the full-open stage by using the MiniBEST Universal RNA Extraction Kit (Code No. 9767, TaKaRa, Dalian, China), in accordance with the manufacturer’s instructions. RNA samples were treated with DNase by using the DNaseI kit (TaKaRa) in accordance with the manufacturer’s instructions prior to reverse transcription and then quantified with a spectrophotometer (Eppendorf, Hamburg, Germany) at 230, 260, and 280 nm.

#### 2.1.2. 3′ Rapid-Amplification of cDNA Ends (RACE)

Total RNA from petal tissue (1 μg) was used to synthesize first-strand complementary DNA (cDNA) with the 3′ full RACE Core Set Ver.2.0 (TaKaRa), in accordance with the manufacturer’s instructions. The 3′ ends of genes were amplified through two rounds of nested PCR with gene-specific primers (Table S1) designed on the basis of the reported conservative regions of *AAAT* and *PPDC* sequences from other plants. First-round PCR was performed by subjecting cDNA to denaturation at 94 °C for 3 min, then to 25 cycles of amplification (94 °C for 30 s, AT for 30 s, and 72 °C for 60 s), and finally to extension at 72 °C for 10 min. The first-round PCR product was used as the template in second-round PCR amplification, which was conducted with 30 cycles under conditions similar to those of first-round PCR.

#### 2.1.3. 5′ Rapid-Amplification of cDNA Ends

Total RNA from petal tissue (1 μg) was used to synthesize first-strand cDNA with the SMARTer^®^ RACE 5′/3′ Kit (Cat. Nos. 634858 and 634859) (TaKaRa), in accordance with the manufacturer’s instructions. Gene-specific primers were designed and synthesized on the basis of the sequenced 3′ ends (Table S1). Then, PCR was performed using the following program: five cycles of amplification (94 °C for 30 s and 72 °C for 3 min), five additional cycles of amplification (94 °C for 30 s, 70 °C for 30 s, and 72 °C for 3 min), and 25 cycles of amplification (94 °C for 30 s, 68 °C for 30 s, and 72 °C for 3 min).

#### 2.1.4. Purification, Cloning, and Sequencing

Isolated PCR products were recovered from 1% agarose gel, purified using the TaKaRa MiniBEST Agarose Gel DNA Extraction Kit Ver.4.0 (TaKaRa), cloned into the PMD18-T vector (TaKaRa) with the pEASY^TM^-T5 Zero Cloning Kit (TransGen Biotech, Beijing, China), and transformed into competent *Escherichia coli* DH5a cells. Recombinant plasmids were extracted using the AxyPrep^TM^ Plasmid Minniprep Kit 50-prep (Axygen, San Francisco, CA, USA). Positive clones were sequenced using the M13 forward and reverse primers with the ABI PRISM^®^ BigDye^TM^ Terminator cycle sequencing kit (Applied Biosystems, Foster City, CA, USA) on the ABI-PRISM 3730 DNA analyzer (Sangon biological engineering technology & services Co., Ltd., Shanghai, China). All sequencing reactions and analyses were repeated at least three times.

### 2.2. Sequence Analysis

Full-length cDNA sequences were analyzed using DNAMAN software (version 5.0, Lynnon Biosoft, Quebec, Canada). A homology search was performed online at the nucleotide level with BLASTn, and at the amino acid level with BLASTp (https://blast.ncbi.nlm.nih.gov/Blast.cgi). A phylogenetic tree was established through the neighbor-joining method with MEGA5.1 program (https://www.megasoftware.net/).

### 2.3 Expression Analysis of RrAAAT and RrPPDC1 Genes in Rosa rugosa

**Figure 2:**
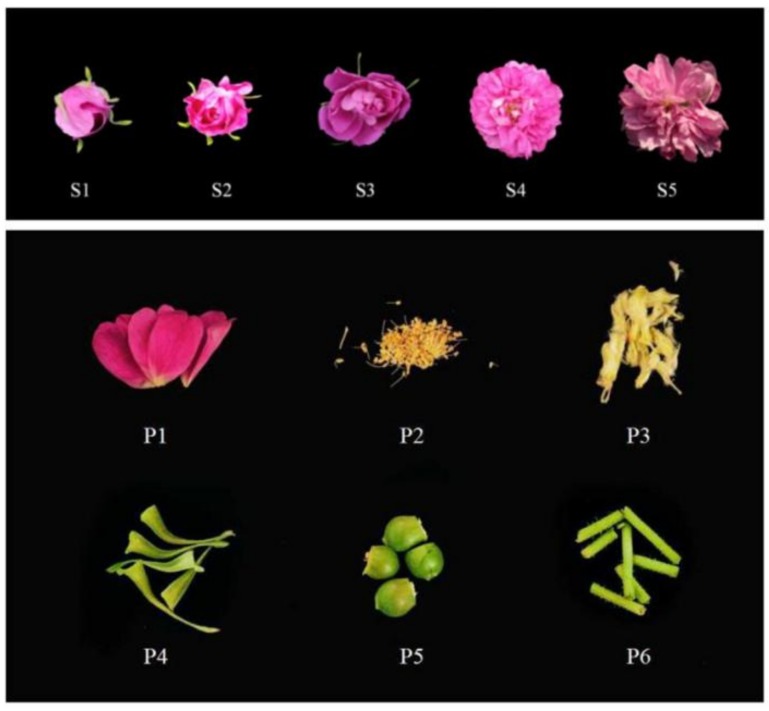
Different developmental stages and different parts of *Rosa rugosa* ‘Tanghong’ flowers. (S1) bud stage; (S2) early-open stage; (S3) half-open stage; (S4) full-open stage; (S5) decline stage; (P1) petal; (P2) stamen; (P3) pistil; (P4) calyx; (P5) receptacle; (P6) pedicle.

The petals of *R. rugosa* ‘Tanghong’ flowers were collected during the bud, early-open, half-open, full-open, and decline stages. The petals, calyx, receptacle, pedicle, stamen, and pistil were gently separated from the full-open flowers (Figure 2). The samples were immediately frozen in liquid nitrogen and stored in the refrigerator at −80 °C for gene expression analysis.

The relative expression levels of *RrAAAT* and *RrPPDC1* mRNA were analyzed through real-time quantitative polymerase chain reaction (RT-PCR) with a BIO-RAD CFX96^TM^ Real-Time System (C1000^TM^ Thermal Cycler) (Bio-Rad, Hercules, California, USA). cDNA was synthesized from 1 μg of RNA by using the PrimeScript^®^ RT reagent Kit with genomics DNA (gDNA) Eraser (TaKaRa). The α-tubulin subunit actin gene from *R. hybrida* (GenBank accession No. AF394915.1) was used as the reference control in real-time quantitative (RT)-PCR analysis. PCR was performed by using the primers listed in Table S2. Quantitative real-time PCR experiments were conducted by using the SMARTe^TM^ RACE cDNA Ampilification Kit (Clontech, Palo Alto, CA, USA), in accordance with the manufacturer’s instructions. Each 25 μL reaction volume contained 12.5 μL of SYBR^®^ Premix Ex Taq^TM^ (2×), 0.5 μL of forward primer, 0.5 μL of reverse primer, 2 μL of cDNA solution as the template, and 9.5 μL of ddH_2_O. The following amplification conditions were applied: initial incubation cycle at 95 °C for 5 min, 40 cycles at 95 °C for 15 s, 53 °C for 34 s, and 72 °C for 40 s. The expression level of each gene was calculated through the 2^−ΔΔCt^ comparative threshold cycle (Ct) method [12]. Ct values were generated by Bio-Rad CFX Manager V1.6.541.1028 software. All experiments were performed in triplicate.

### 2.4. Construction of the RrAADC, RrAAAT, and RrPPDC1 Overexpression Vectors

The first strand of the reverse-transcribed cDNA was synthesized in accordance with the specifications of the PrimeScript^TM^ first strand cDNA Synthesis Kit. Upstream and downstream primers for the in vitro cloning of the target gene were designed on the basis of the full-length cDNA sequences of *RrAADC* (KJ027532, NCBI), *RrAAAT* (MG820126, NCBI), and *RrPPDC1* (KY622034, NCBI). The cross-region of the primers should contain a complete open reading frame (ORF) sequence (Table S3). PCR was performed by subjecting cDNA to denaturation at 94 °C for 3 min, and then to 25 cycles of amplification (94 °C for 30 s, AT for 30 s, and 72 °C for 120 s), and finally to extension at 72 °C for 10 min. The recombinant plasmids pCAMBIA1304–*RrAADC*, pCAMBIA1304–*RrAAAT*, and pCAMBIA1304–*RrPPDC1* were constructed by using pCAMBIA1304 with *Nco*I and *BstE*II restriction endonuclease pCAMBIA1304 expression vectors, in accordance with the specifications of the In-Fusion HD Cloning Kit (Clontech). The restriction enzymes and primers used in this experiment are listed in Table S4. The constructed pCAMBIA1304–*RrAADC*, pCAMBIA1304–*RrAAAT*, and pCAMBIA1304–*RrPPDC1* plasmids were introduced into *Agrobacterium tumefaciens* EHA105 through electroporation, and identified through PCR.

### 2.5. Plant Genetic Transformation and Phenotypic Analysis 

The recombinant *Agrobacterium* plasmid was activated and then transformed into *Petunia hybrida* (Mitchell diploid, MD) via *Agrobacterium*-mediated genetic transformation [13]. Hygromycin selection pressures were set as 7 and 6 mg/L during callus differentiation and rooting, respectively, and carbenicillin selective pressure was set as 500 mg/L. Tissue-cultured petunia seedlings were transferred to artificial climate boxes after growing five leaves with one sprout and cultivated under the light/dark period of 16 h/8 hr, light intensity of 200 µmol m^−2^s^−1^, temperature of 25 °C/23 °C, and relative humidity of 70%.

DNA was extracted from the leaves of established petunia plants by using the MiniBEST Plant Genomic DNA Extraction Kit (TaKaRa) in accordance with the manufacturer’s directions. Then, transgenic plants were subjected to PCR analysis. The morphological characteristics of the wild-type and transgenic *Petunia* plants, such as plant growth and floral size, were observed. Gas chromatography with mass spectrometry (GC–MS) was used to analyze the aromatic compounds and contents of blooming wild-type and transgenic *Petunia* flowers. *RrAADC*, *RrAAAT*, and *RrPPDC1* expression levels in *Petunia* flowers were detected through semiquantitative PCR with the primers listed in Table S4. The ubiquitin gene was used as the reference control and was amplified using the following primer pairs: ubiquitin F(5′-TGGAGGATGGAAGGACTTTGG-3′) and ubiquitin R(5′-CAGGACGACAACAAGCAACAG-3′). The thermal program was as follows: 3 min at 94 °C, 30 cycles of 30 s at 94 °C, 30 s at ambient temperature, and 60 s at 72 °C, and a 10 min extension at 72 °C.

### 2.6. Gas Chromatography with Mass Spectrometry Analysis of Floral Volatiles from Transgenic and Control Petunia Plants

One blooming petunia flower was collected, weighed, and immediately placed in 10 mL sample vials. The internal standard (3-nonanone, 0.8 μg·μL^−1^, Sigma Aldrich, Saint Louis, MO, USA) was added, and the vials were immediately sealed with septa (PTFE-butyl synthetic rubber). The extraction and concentration of the floral headspace volatiles from the samples were performed in accordance with the headspace solid-phase microextraction method (manual headspace sampler with a 100 μm polydimethylsiloxane (PDMS) fiber, (Supelco, Bellefonte, PA, USA). The sample vials were heated in a water bath at 40 °C for 40 min. Three replicates were prepared for each transgenic and control plant.

GC-MS analysis was performed using Trace DSQ (Thermo Fisher Scientific, Waltham, MA, USA) [11]. The mass spectral ionization temperature was set as 250 °C. The electron energy was 70 eV. Mass spectra were collected through automatic scanning at *m*/*z* 30–600 amu. The flow rate of the helium (99.99%) carrier gas on the free fatty acid phase (FFAP) elastic quartz capillary vessel column (60 m × 0.32 mm I.D., 1.0 μm film), (Agilent, Santa Clara, CA, USA) was 1.00 mL/min. Then, the sample was injected under splitless mode for 2 min at an injector temperature of 250 °C. The column temperature was programmed as follows: The initial temperature was maintained at 50 °C for 1 min, increased to 120 °C at 5 °C/min, then increased to 200 °C at 8 °C/min, and finally increased to 250 °C at a rate of 12 °C/min, which was maintained for 7 min.

The qualitative and quantitative analyses of floral volatiles were conducted using Xcalibur software (Thermo Fisher Scientific). Qualitative analysis was performed as follows: Spectrometric data were compared with the mass spectral data obtained from the United States National Institute of Standards and Technology mass spectral library [14] and the Wiley Registry™ of mass spectral data [15]. Mass spectra were manually resolved and confirmed through the comparison of the Kovat’s retention indices retrieved from relevant reports. Only results with positive and negative matching values of more than 800 (maximum: 1000) were selected and analyzed.

Quantitative analysis was performed with 3-nonanone (0.8 μg·μL^−1^) as the internal standard. The selected ion monitoring technique was used for the quantitative analysis of headspace compounds, which were quantified through the response factor method combined with the internal standard method. Mass fractions were calculated using the following formula: Content of each component (μg·g^−1^) = (Peak area of each component/peak area of internal standard × Concentration of internal standard (μg·μL^−1^) × Volume of internal standard/Sample weight (g)).

## 3. Results

### 3.1. RrAAAT and RrPPDC1 Complementary DNA Isolation, Sequence Analysis, and Phylogenetic Tree

We successfully obtained *RrAAAT* and *RrPPDC1* cDNA through the RACE method by using RNA extracted from the petals of blooming *R. rugosa* ‘Tanghong’ as the template. The full length of *RrAAAT* cDNA is 1466 bp. The *RrAAAT* cDNA sequence encodes 422 amino acids, including an initiation codon, a complete open reading frame (1266 bp), a termination codon, a 3′ noncoding region (200 bp), and a polyA tail (11 bp). The GenBank accession number of this sequence is MG820126. The *RrPPDC1* cDNA sequence has a full length of 2078 bp, and it encodes 606 amino acids, including an initiation codon, a complete open reading frame (1818 bp), a termination codon, a 5′ noncoding region (71 bp), a 3′ noncoding region (177 bp), and a polyA tail (12 bp). The GenBank accession number of this sequence is KY622034 (Figure S1).

**Figure 3:**
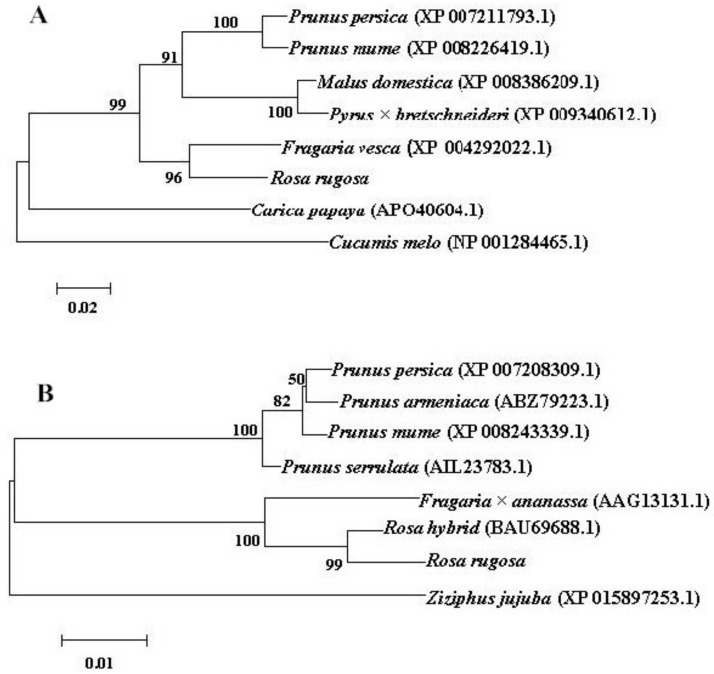
Homology analysis of *RrAAAT* (**A**) and *RrPPDC1* (**B**) gene sequences with NCBI BLAST. The numbers in the cladogram show homology percentage; accession numbers are given.

A NCBI homology search indicates that the sequence of *RrAAAT* shares 87%, 87%, 93%, 86%, and 86% homology with the sequences of *AAAT* from *Prunus persica* (AB305070.1), *Prunus mume* (XP 008226419.1), *Malus domestica* (XP 008386209.1), *Pyrus* × *bretschneideri* (XP 009340612.1), and *Fragaria vesca* (XP 004292022.1), respectively. The sequence of *RrPPDC1* shares 92%, 92%, 99%, 97%, and 92% homology with the sequences of *PPDC1* from *Prunus serrulata* (AIL23783.1), *P. persica* (XP 007208309.1), *R. hybrida* (BAU69688.1), *Fragaria* × *ananassa* (AAG13131.1), and *P. mume* (XP 008243339.1), respectively (Figure 3).

### 3.2. Temporal and Spatial Expression Analyses of RrAAAT and RrPPDC1 Genes in Rosa rugosa

#### 3.2.1. Analysis of *RrAAAT* and *RrPPDC1* Expression Patterns in *Rosa rugosa* at Different Development Stages 

**Figure 4:**
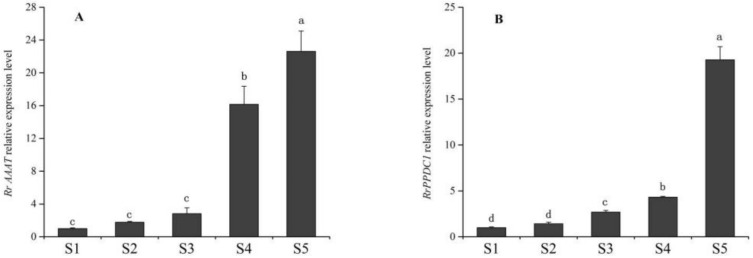
Quantification of gene expression levels in different stages of floral development of *R. rugosa* ‘Tanghong’ through real-time PCR. Values represent the means ± standard error (SE); Different letters (a, b, c) denote significant differences (Least Significant Difference (LSD) test, *p* ≤ 0.05). (**A**) relative expression level of *RrAAAT* gene; (**B**) relative expression level of *RrPPDC1* gene; (S1) bud stage; (S2) early-open stage; (S3) half-open stage; (S4) full-open stage; (S5) decline stage.

As shown in Figure 4, *RrAAAT* expression increased continuously during blooming, and peaked during senescence. *RrAAAT* expression increased slowly from the bud stage to the half-open stage and was 5.7-fold higher during the full-open stage than during the half-open stage. *RrAAAT* expression continuously increased during the decline stage (Figure 4A). The trend followed by the change in *RrPPDC1* expression was similar to that followed by the change in *RrAAAT* expression; that is, *RrPPDC1* expression smoothly increased from the bud stage to the full-open stage. *RrPPDC1* expression, however, rapidly increased and peaked, and was 4.47-fold higher during the decline stage than during the full-open stage (Figure 4B).

#### 3.2.2. Analysis of *RrAAAT* and *RrPPDC1* Expression in Different Parts of Flower Organs in *Rosa rugosa*

**Figure 5:**
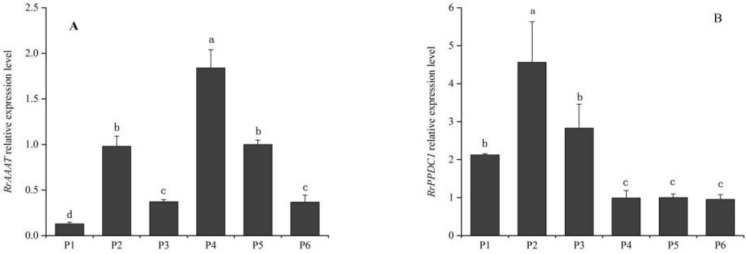
Quantification of gene expression levels in different parts of flower organs of *R. rugosa* ‘Tanghong’ through real-time PCR. (**A**) Relative expression level of *RrAAAT* gene; (**B**) relative expression level of *RrPPDC1* gene; (P1) petal; (P2) stamen; (P3) pistil; (P4) calyx; (P5) receptacle; (P6) pedicle.

As shown in Figure 5, the highest level of *RrAAAT* was observed in the calyx, followed by that in the stamen and receptacle. We observed the lowest *RrAAAT* expression level in the petals (Figure 5A). *RrPPDC1* expression in the stamen was higher than that in the pistil and the petals, and was low in the calyx, receptacle, and pedicel (Figure 5B).

### 3.3. Construction of the Overexpression Vectors, Plant Genetic Transformation, and Phenotypic Analysis 

We successfully constructed the recombinant plasmids pCAMBIA1304–*RrAADC*, pCAMBIA1304–*RrAAAT*, and pCAMBIA1304–*RrPPDC1* by using pCAMBIA1304 expression vector with *Nco*I and *BstE*II restriction endonuclease. After enzyme digestion and sequencing verification (Figure S2), the constructed pCAMBIA1304–*RrAADC*, pCAMBIA1304–*RrAAAT*, and pCAMBIA1304–*RrPPDC1* plasmids were introduced into *A. tumefaciens* EHA105 through electroporation, and identified through PCR (Figure S3).

#### 3.3.1. *RrAADC* and *RrAAAT* Overexpression Increases the Content of 2-Phenylethanol in *Petunia*

After hygromycin selection, we transplanted the transgenic and control *Petunia* plants to a climate box. We cultivated the plants under the same environmental conditions. We randomly selected five *RrAADC* transgenic plants and four *RrAAAT* transgenic plants established for DNA detection. A band with a size of approximately 300 bp was amplified from four and three plants respectively, but not from the control plants (Figure S4). The size of this band was consistent with that of the positive control band. This finding indicates that *RrAADC* and *RrAAAT* gene was integrated into the *Petunia* genome respectively. 

**Figure 6:**
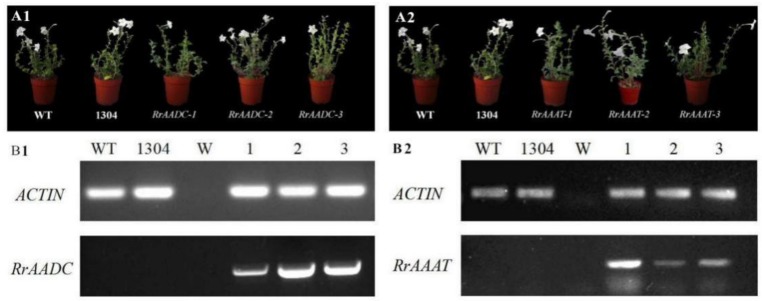
Phenotype and RT-PCR analysis of wild-type and *RrAADC* and *RrAAAT* transgenic *Petunia*. (**A1**,**A2**) Plant morphology of *Petunia* plant after 150 days of transplanting; (**B1**,**B2**) messenger RNA (mRNA) expression of *RrAADC* and *RrAAAT* in the flowers of transgenic *Petunia* plants; (WT) wild type; (1304) pCAMBIA1304 transgenic plants; (W) Water; (1–3) transgenic plants.

After hygromycin selection and PCR detection, three *RrAADC*-overexpressing plants and three *RrAAAT*-overexpressing plants were randomly selected and designated as *RrAADC-1*, *RrAADC-2*, *RrAADC-3* (Figure 6(A1)), and *RrAAAT-1*, *RrAAAT-2*, *RrAAAT-3* (Figure 6(A2)) for follow-up phenotype observation and analysis. After flowering, RT-PCR analysis were conducted with flowers as the test materials. No bands were amplified from the wild-type and control plants. By contrast, bands were amplified from all transgenic plants. These results indicate that *RrAADC* and *RrAAAT* genes were correctly transcribed, low gene expression was detected in the *RrAADC-1* transgenic line (Figure 6(B1)), and high gene expression level was detected in the *RrAAAT-1* line (Figure 6(B2)). The floral shapes and sizes of *RrAADC* and *RrAAAT* transgenic plants were not significantly different from those of the control plants.

We adopted headspace solid-phase microextraction methods combined with GC–MS, to determine the differences between the aromatic components of transgenic and control *Petunia* flowers. We selected eight main representative aromatic components that were present at high contents (i.e., methyl benzoate, benzyl benzoate, isoeugenol, benzyl tiglate, 2-phenylethanol, eugenol, benzyl alcohol, benzoic acid, and 2-phenylethyl ester) for statistical analysis. 

**Figure 7:**
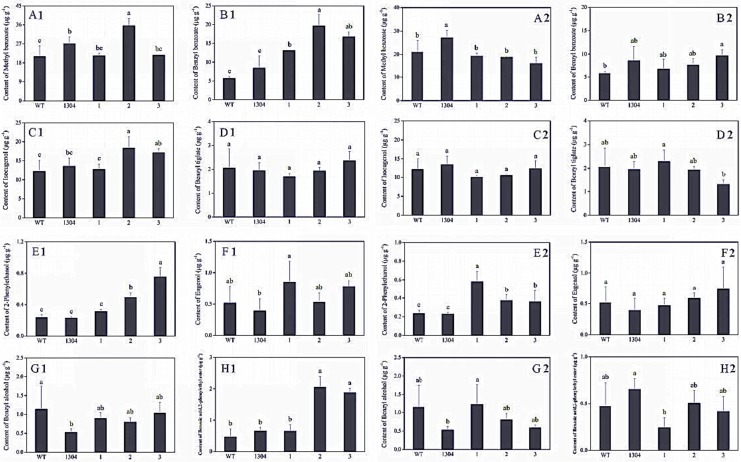
Analysis of the main volatile components in flower of *Petunia*. (A1–H1) Main volatile components of *RrAADC* transgenic and control plants; (A2–H2) main volatile components of *RrAAAT* transgenic and control plants; (**A**) methyl benzoate; (**B**) benzyl benzoate; (**C**) isoeugenol; (**D**) benzyl tiglate; (**E**) 2-phenylethanol; (**F**) eugenol; (**G**) benzyl alcohol; (**H**) benzoic acid, 2-phenylethyl ester; (WT) wild type; (1304) pCAMBIA1304 transgenic plants; (1–3) transgenic plants. (±SE, n = 3).

We found that the benzyl benzoate (Figure 7(B1)), 2-phenylethanol (Figure 7(E1)), benzoic acid, 2-phenylethyl ester (Figure 7(H1)) contents of *RrAADC* transgenic plants were higher than those of the control plants. The 2-phenylethanol contents of the *RrAADC-1*, *RrAADC-2*, and *RrAADC-3* plants were 1.4-, 2.1-, and 3.23-fold higher than those of the wild-type plants, respectively (Figure 7(E1)). The methyl benzoate contents of the *RrAADC-2* plants were 1.7- and 1.29-fold higher those of that the wild-type and transgenic pCAMBIA1304 vector plants, respectively (Figure 7(A1)). The volatile benzyl benzoate contents of the *RrAADC-1*, *RrAADC-2*, and *RrAADC-3* plants were 2.3-, 3.4-, and 2.92-fold higher than those of the wild-type plants, respectively, and were 1.5-, 2.3-, and 1.99-fold higher than those of the transgenic pCAMBIA1304 vector plant, respectively (Figure 7(B1)). The isoeugenol contents of *RrAADC-2*- and *RrAADC-3*-overexpressing plants were 1.5- and 1.4-fold higher than those of the wild-type plants, respectively, and 1.36- and 1.26-fold higher than those of the transgenic pCAMBIA1304 vector plant, respectively (Figure 7(C1)). The benzoic acid, 2-phenylethyl ester contents of the *RrAADC-2* and *RrAADC-3* plants were 4-fold higher than those of the wild-type plants (Figure 7(H1)). The other aromatic components of the *RrAADC* transgenic plants did not significantly differ from those of the control plants.

Similarly, we analyzed eight main aromatic components, and found that the 2-phenylethanol content of *RrAAAT* transgenic *Petunia* flowers increased. Specifically, the 2-phenylethanol contents of the *RrAAAT-1*, *RrAAAT-2*, and *RrAAAT-3* lines were 2.52-, 1.59-, and 1.53-fold higher than those of the wild-type plants, respectively (Figure 7(E2)). The methyl benzoate contents of transgenic plants were slightly lower than those of the wild-type and transgenic pCAMBIA1304 plants (Figure 7(A2)). The benzyl benzoate content of the *RrAAAT-3* line was 1.68- and 1.1-fold higher than those of the wild-type and transgenic pCAMBIA1304 plants. By contrast, the benzyl benzoate contents of *RrAAAT-1* and *RrAAAT-2* lines were slightly lower than those of pCAMBIA1304 transgenic plants (Figure 7(B2)). The contents of the other aromatic components of *RrAAAT* transgenic plants negligibly differed from those of the control plants.

#### 3.3.2. *RrPPDC1* Overexpression Increases the Diameter of *Petunia* Flowers

**Figure 8:**
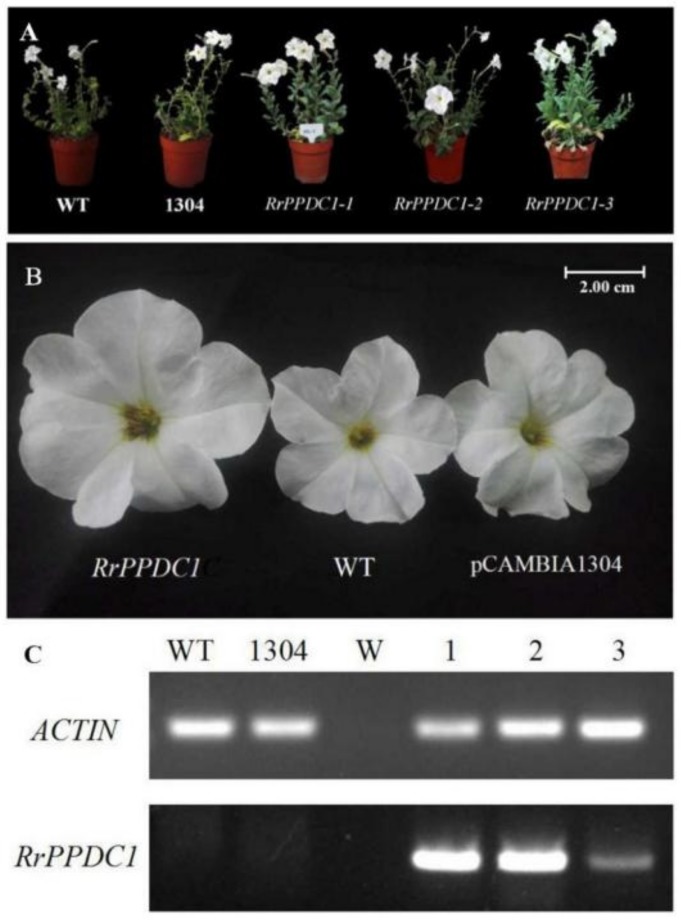
Phenotype and RT-PCR analysis of wild-type and *RrPPDC1* transgenic *Petunia*. (**A**,**B**) Plant morphology of *Petunia* plants after 150 days of transplanting; (**C**) mRNA expression of *RrPPDC1* in the flowers of transgenic and control *Petunia* plants; (WT) wild type; (1304) pCAMBIA1304 transgenic plants; (W) Water; (1–3) transgenic plants.

After hygromycin selection and PCR detection (Figure S4), three *RrPPDC1*-overexpressing plants were randomly selected for follow-up phenotype observation and analysis, and designated as *RrPPDC-1*, *RrPPDC-2*, and *RrPPDC-3* (Figure 8A). We subjected the flowers of the selected plants to RT-PCR analysis. No bands were amplified from the wild-type and control plants, whereas bands were amplified from the three transgenic plants. These results indicated that the foreign gene was correctly transcribed, and that the *RrPPDC1* expression levels in the *RrPPDC1-3* transgenic lines had decreased (Figure 8C). We regularly observed the *Petunia* plants for morphological changes, and found that the corolla diameters of the *RrPPDC1* transgenic lines were larger than those of the control plants (Figure 8B, Table 1).

**Figure 9:**
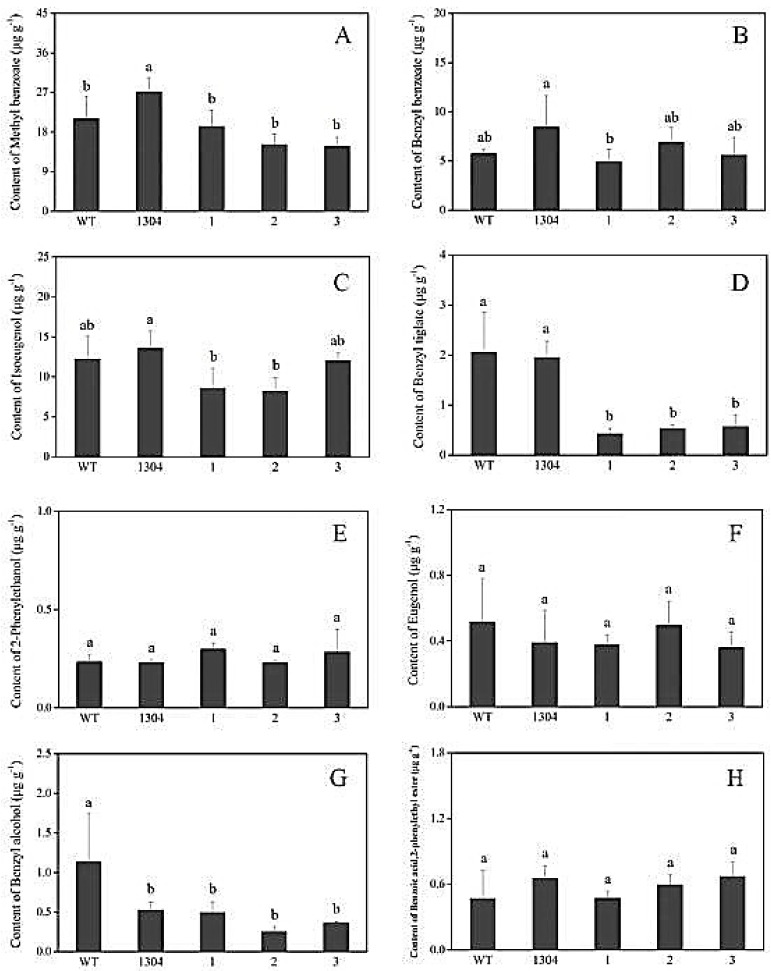
Analysis of the main volatile components in flower of *RrPPDC1* transgenic and control *Petunia*. (**A**) methyl benzoate; (**B**) benzyl benzoate; (**C**) isoeugenol; (**D**) benzyl tiglate; (**E**) 2-phenylethanol; (**F**) eugenol; (**G**) benzyl alcohol; (**H**) benzoic acid, 2-phenylethyl ester; (WT) wild type; (1304) pCAMBIA1304 transgenic plants; (1–3) *RrPPDC1* transgenic plants (±SE, n = 3).

Our GC–MS test results revealed that the 2-phenylethanol content of *RrPPDC1* transgenic *Petunia* flowers negligibly differed from that of the wild-type flowers (Figure 9E). The methyl benzoate and isoeugenolin contents of the transgenic plants were slightly lower than those of the control plants (Figure 9A,C). The benzyl tiglate contents of the *RrPPDC1* transgenic plants were lower than those of the control plants, and were three-quarters that of the wild-type plants (Figure 9D,G). The contents of the other aromatic components of the transgenic plants negligibly differed from those of the control plants.

## 4. Discussion

Aroma is an important quality characteristic of horticultural plants. The production of aromatic compounds is closely related to the ontogeny period of the plant, and is regulated by a series of genes. In general, the expression of aroma-related genes mainly peaks during blooming, and then begins to decline rapidly [16]. We previously showed that the 2-phenylethanol content of *R. rugosa* petals increased gradually from the bud stage to the decline stage, and peaked during the decline stage. 2-Phenylethanol was not detected during the bud stage. In this study, we found that the expression levels of *RrAAAT* and *RrPPDC1* in *R. rugosa* petals increased and peaked during the decline stage. This expression pattern coincides with the 2-phenylethanol release pattern during rose development. The consistency between the expression patterns of *RrAAAT* and *RrPPDC1* and the release pattern of 2-phenylethanol, in turn, indicates that *RrAAAT* and *RrPPDC1* may be closely related to the synthesis of 2-phenylethanol in traditional Chinese roses.

In *R. rugosa*, petals are the main floral organs that release fragrance and that have the highest 2-phenylethanol content [11]. However, we found that *RrAAAT* and *RrPPDC1* were expressed at low levels in petals in this study. Specifically, we observed the lowest *RrAAAT* expression level in petals. This finding indicates that the expression levels of *RrAAAT* and *RrPPDC1* in different floral organs were not positively correlated with the release of 2-phenylethanol. Similarly, the expression levels of two genes, *LePAR1* and *LePAR2*, related to 2-phenylethanol synthesis in different tomato organs are not positively correlated with the release of 2-phenylethanol, and that 2-phenylethanol is mainly released in the fruit, despite the highest expression levels of *LePAR1* and *LePAR2* being found in the bud [17]. In *R. chinensis*, *RyPPDC* expression in petals is also lower than those in other organs [10]. This phenomenon may account for the inconsistency of 2-phenylethanol synthesis and release. In *R. rugosa*, 2-phenylethanol may be synthesized in other floral organs, and then transported to petals for release. Further experimental verification is needed to confirm the mechanisms underlying the synthesis and transport of 2-phenylethanol in *R. rugosa*.

In *R. chinensis*, L-phenylalanine is converted into phenylacetaldehyde under the action of *AADC*. In addition, phenylacetaldehyde synthase (*PAAS*) in *Petunia* and aromatic aldehyde synthase (*AAS*) in *Arabidopsis thaliana* can also catalyze the formation of phenylacetaldehyde [18,19]. However, *AADC* in tomato converts l-phenylalanine into phenylethylamine, and the overexpression of the gene family of *AADC* results in the release of 2-phenylacetaldehyde and 2-phenylethanol in transgenic fruits at volumes that are higher than those in wild-type plants [20]. In this study, we found that in addition to 2-phenylethanol contents, benzyl benzoate, benzoic acid, and 2-phenylethyl ester contents in *RrAADC* transgenic *Petunia* increased. The specific reason underlying this increase, however, is unclear. In future works, RNA-seq technology should be used to compare the gene expression patterns of wild-type and transgenic plants, and to explore the molecular mechanism underlying the phenotypic changes. Plant metabolites mainly originate from three aromatic amino acids: tyrosine, tryptophan, and phenylalanine [21]. l-phenylalanine is preferentially used as the substrate in the metabolism of 2-phenylethanol, under the mediation of *RyAAAT* in *R. hybrida* “Yves Piaget” and *CmArAT* in *Cucumis melo* [10,22]. In our study, *RrAAAT* overexpression likely increased the 2-phenylethanol content of *Petunia* flowers by promoting phenylalanine conversion and 2-phenylethanol synthesis. The *PPDC* gene participates not only in the metabolism of 2-phenylethanol in *R. hybrida*, but also in the seasonal release of floral fragrance [10]. We found that *RrPPDC1* overexpression negligibly changed the content of the floral fragrance components of *Petunia*, given the absence of the necessary substrate in *Petunia*. However, the floral size of transgenic *RrPPDC1 Petunia* drastically increased. This phenotypic change should be investigated in future studies.

## 5. Conclusions

In this study, *RrAADC*, *RrAAAT*, and *RrPPDC1* genes were cloned from *R. rugosa* and introduced into *Petunia* to verify their functions. In transgenic *Petunia* flowering lines, *RrAADC* and *RrAAAT* overexpression promoted 2-phenylethanol synthesis and accumulation and increased 2-phenylethanol content. These results indicate that the content of 2-phenylethanol can be altered by manipulating the expression of a single gene. The findings of this study provide the basis for the introduction of genes related to 2-phenylethanol synthesis into roses to increase the 2-phenylethanol content of rose essential oil. 

## Figures and Tables

**Table 1:** The corolla diameters of *RrPPDC1* transgenic and control *Petunia* flowers (mm).

WT	pCAMBIA1304	*RrPPDC1-1*	*RrPPDC1-2*	*RrPPDC1-3*
49.43 ± 2.29 c	49.00 ± 2.60 c	56.14 ± 1.14 a	53.56 ± 0.47 b	55.34 ± 1.75 ab

Values represent the means ±SE; Different letters (a, b, c) denote significant differences (LSD test, *p* ≤ 0.05).

## Supplementary Materials

The following are available online at http://www.mdpi.com/2073-4425/9/12/576/s1. Figure S1: Nucleotide sequence and amino acid sequence of *RrAAAT* and *RrPPDC1*, Figure S2: Enzyme digestion verification of the recombinant plasmid of the overexpression vectors, Figure S3: Colony PCR verification of *Agrobacterium* transformants, Figure S4: DNA detection of transgenic *Petunia* plants, Table S1: Primers used for the isolation of *RrAAAT* and *RrPPDCs* genes from *R. rugosa*, Table S2: Gene-specific primers sequence for detection by RT-PCR, Table S3: The primers used for amplifying the open reading frames of the *RrAADC*, *RrAAAT* and *RrPPDC1* genes, Table S4: The primers used for enzyme digestion.
