# Supplementary material for: Cloning and Functional Verification of Genes Related to 2-Phenylethanol Biosynthesis in Rosa rugosa
Source: Genes (Basel). 2018 Nov 26;9(12):576. doi: 10.3390/genes9120576 (PMC6316838; doi:10.3390/genes9120576)
Supplement: Supplementary file 1 [file genes-09-00576-s001.pdf]

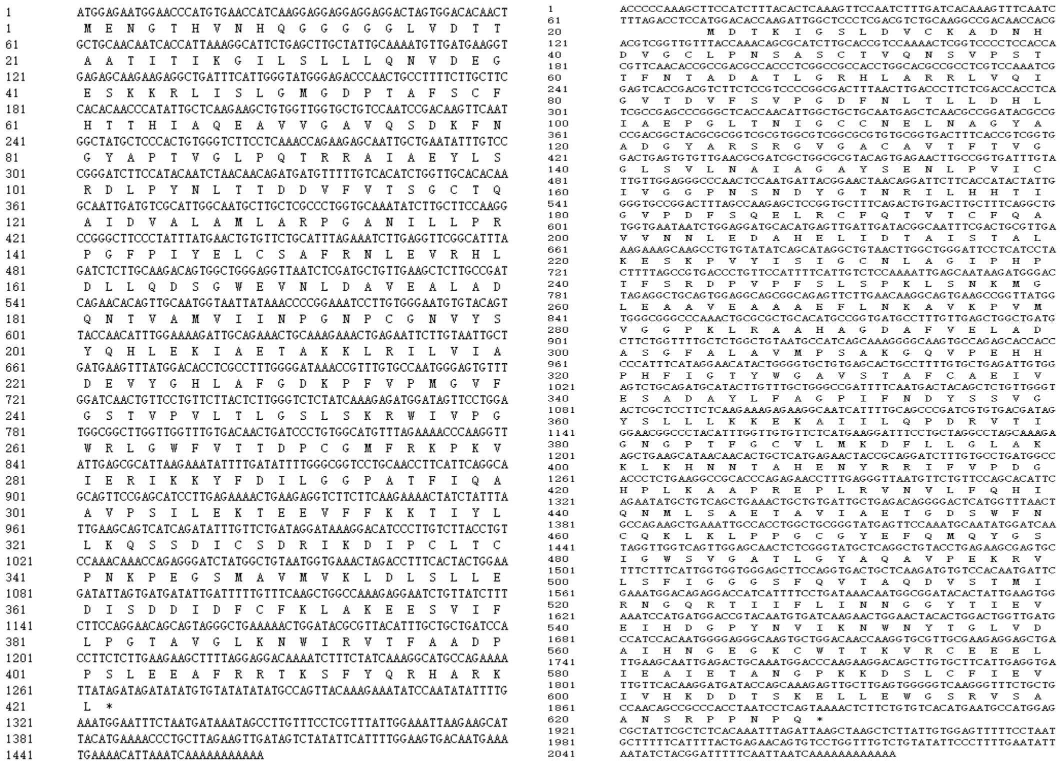

Figure S1. Nucleotide sequence and amino acid sequence of *RrAAAT* and *RrPPDC1.* (**A**) *RrAAAT*, GenBank accession No.MG820126; (**B**) *RrPPDC1*, GenBank accession No. KY622034.

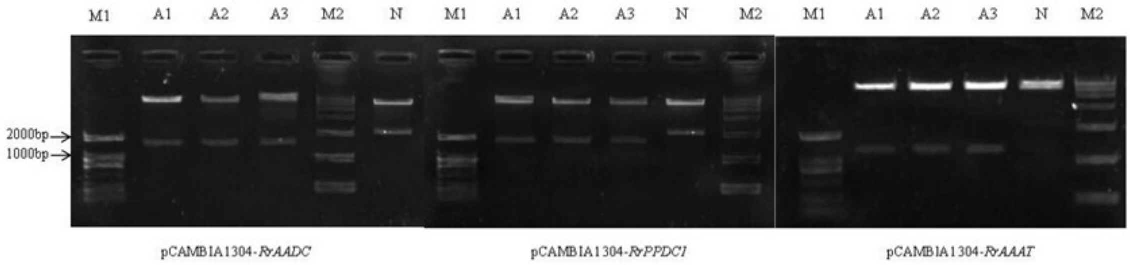

Figure S2. Enzyme digestion verification of the recombinant plasmid of the overexpression vectors. (M1) Marker DL 2000; (M2) Marker DL 15000; (A1-A3) the result of digestion. (N) negative control.

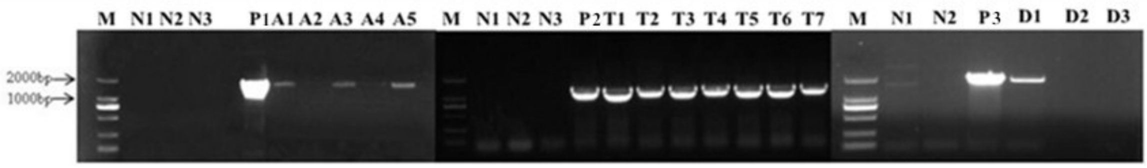

Figure S3. Colony PCR verification of *Agrobacterium transformants*. (M) Marker DL 2000; (N1) ddH$_2$O as template; (N2) Agrobacterium EHA liquid as template;. (N3) YEB liquid medium as template; (P1) pCAMBIA1304-*RrAADC* as template;. (A1-A5) Agrobacterium EHA of pCAMBIA1304-*RrAADC* as template; (P2) pCAMBIA1304-*RrAAAT* as template; (T1-T7) Agrobacterium EHA of pCAMBIA1304-*RrAAAT* as template; (P3) pCAMBIA1304-*RrPPDC1* as template; (D1-D3) Agrobacterium EHA of pCAMBIA1304-*RrPPDC1* as template.

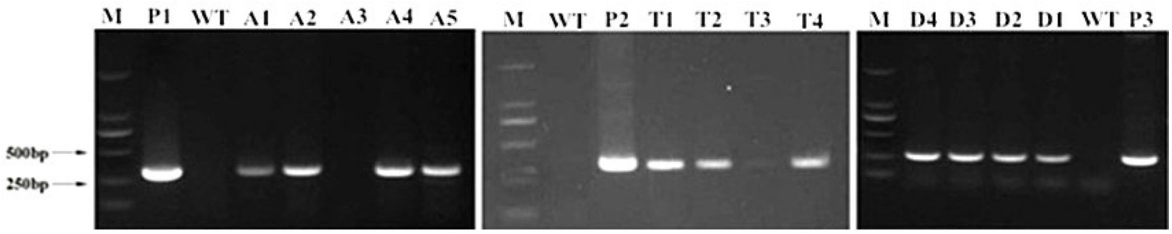

Figure S4. DNA detection of transgenetic *Petunia* plants. (M) Marker DL2000; (WT) wild type; (P1) pCAMBIA1304-*RrAADC* plasmid; (P2) pCAMBIA1304-*RrAAAT* plasmid; (P3) pCAMBIA1304-*RrPPDC1* plasmid; (A1-A5) *RrAADC* overexpressing resistant plants; (T1-T4) *RrAAAT* overexpressing resistant plants; (D1-D4) *RrPPDC1* overexpressing resistant plants.

Table S1. Primers used for isolation of *RrAAAT* and *RrPPDCs* genes from *R. rugosa*

Gene	Oligonucleotide sequence (5′-3′)	Application	Annealing Temperature (Ta, ℃)	Time (s)
RrAAAT	AAATCCTTGTGGGAATGTG	1st of 3′ RACE	50	60
RrAAAT	ACCCAAGGTTATTGAGCG	2nd of 3′ RACE	52	60
RrAAAT	GAGCATAGCCATTGAACTTGTCGGATTG	5′ RACE	65	120
RrPPDC1	GACTTATTGGGGTGCTGTG	1st of 3′ RACE	54	60
RrPPDC1	GTCAGTCGGTGCTACTCTCG	2nd of 3′ RACE	57	60
RrPPDC1	CGGCGATTATGGCAGTGTTTGGGGT	5′ RACE	63	120

Table S2. Gene-specific primers sequence for detection by real-time quantitative RT-PCR

Gene	Oligonucleotide sequence (5′-3′)	Melting Temperature (Tm, ℃)	Time (s)
Actin-F	TGAGGCCATTTACGACAT	53	30
Actin-R	AGATCACAGGAGCATAGGAG	53	30
RrAAAT-F	TGATCCCTGTGGCATGTTTA	53	30
RrAAAT-R	TCTCAAGGATGCTCGGAACT	53	30
RrPPDC1-F	GCTTTCAGGCTGTGGTGAAT	53	30
RrPPDC1-R	GGAATCCCAGCCAAGTTACA	53	30

Table S3. The primers used for amplifying the open reading frames of *RrAADC*, *RrAAAT* and *RrPPDC1* gene

Primer	Oligonucleotide sequence (5′-3′)	Annealing temperature (Ta, °C)	Time (s)
RrAADC-F	TCACCTAGCTGTGTTCAACT	55	120
RrAADC-R	TGGTACGTACAAGTAGTTT	55	120
RrAAAT-F	ATGGAGAATGGAACCCATGTG	53	120
RrAAAT-R	CTATAATTTTCTGGCATGCCTT	53	120
RrPPDC1-F	TACATGGAACCCTCTACACTCA	57	120
RrPPDC1-R	CAACTTTGATTCTTTCAGCTCCG	57	120

Table S4. The primers used for enzyme digestion

Primer	Oligonucleotide sequence (5′-3′)	The sites of restriction endonuclease
RrAADC-F	GGACTCTTGACCATGGTTTCACCTAGCTGTGTTCAACT	*Nco* I
RrAADC-R	ATTCGAGCTGGTCACCTGGTACGTAGTACAAGTAGTTT	*BstE* II
RrAAAT-F	GGACTCTTGACCATGGTTATGGAGAATGGAACCCATGTG	*Nco* I
RrAAAT-R	TCAGATCTACCATGGCTATAATTTTCTGGCATGCCTT	*Nco* I
RrPPDC1-F	GGACTCTTGACCATGGTTTACATGGAACCCTCTACACTCA	*Nco* I
RrPPDC1-R	ATTCGAGCTGGTCACCCAACTTTGATTCTTTCAGCTCCG	*BstE* II
